# Heart Failure and Gut Microbiota: What Is Cause and Effect?

**DOI:** 10.34133/research.0610

**Published:** 2025-02-20

**Authors:** Shichun Shen, Beiduo Tian, Haizhu Zhang, Yu-Chen Wang, Tao Li, Yang Cao

**Affiliations:** ^1^ Department of Cardiology, The First Affiliated Hospital of USTC, Division of Life Sciences and Medicine, University of Science and Technology of China, Hefei, Anhui 230001, China.; ^2^ School of Basic Medical Sciences, Division of Life Sciences and Medicine, University of Science and Technology of China, Hefei, Anhui 230027, China.; ^3^ Department of Medicine, Division of Cardiology, Department of Microbiology, Immunology and Molecular Genetics, and Department of Human Genetics, University of California, Los Angeles, CA, USA.; ^4^ Department of Anesthesiology, Laboratory of Mitochondrial Metabolism and Perioperative Medicine, National Clinical Research Center for Geriatrics, West China Hospital of Sichuan University, Chengdu, Sichuan, China.

## Abstract

Emerging evidence highlights the central role of gut microbiota in maintaining physiological homeostasis within the host. Disruptions in gut microbiota can destabilize systemic metabolism and inflammation, driving the onset and progression of cardiometabolic diseases. In heart failure (HF), intestinal dysfunction may induce the release of endotoxins and metabolites, leading to dysbiosis and exacerbating HF through the gut–heart axis. Understanding the relationship between gut microbiota and HF offers critical insights into disease mechanisms and therapeutic opportunities. Current research highlights promising potential to improve patient outcomes by restoring microbiota balance. In this review, we summarize the current studies in understanding the gut microbiota–HF connection and discuss avenues for future investigation.

## Introduction

The gut microbiota refers to the diverse community of microorganisms residing in the host’s intestines. These microbes play crucial roles in various physiological processes and produce metabolites that enter the bloodstream, influencing the host’s health [1]. Disruptions in the host’s physiological functions or internal homeostasis can disturb this balance, leading to gut microbiota dysregulation and triggering pathological changes in the host.

Heart failure (HF) is a cardiovascular disease characterized by diminished cardiac output and ranks among the leading causes of mortality worldwide. Chronic HF patients frequently experience intestinal dysfunction, including structural abnormalities and increased permeability, which facilitate the translocation of gut microbiota, endotoxins, cytokines, and metabolites [2]. For example, lipopolysaccharide, a potent pro-inflammatory molecule released by gram-negative bacteria, is elevated in HF patients compared to that in healthy individuals and plays a critical role in the systemic inflammation associated with HF. In this review, we explore the interactions between gut microbiota composition, microbial secretions, and HF, providing new perspectives on diagnosis, treatment, and future research directions for HF.

## Composition and Function of the Gut Microbiota

The gut microbiota, consisting of over 1,000 bacterial species within the human gastrointestinal tract, forms a symbiotic relationship with the host and plays crucial roles in metabolism, immune function, and overall health [3].

The gut microbiota plays a pivotal role in maintaining intestinal and overall host health by producing and releasing various secretions. These microbial secretions, including metabolites and bacteriocins, regulate immune responses by activating pattern recognition receptors and adhering to epithelial surfaces, thus influencing disease onset and progression [4]. Beyond bacterial contributions, gut-resident viruses and fungi also affect immune responses and contribute to central nervous system disorders, gastrointestinal diseases, and metabolic syndromes [5,6]. For example, Coxsackievirus promotes the progression of dilated cardiomyopathy and HF by cleaving immune-response-related host proteins and activating mitochondrial dysfunction-mediated pathways that lead to cell death [7]. Formylmethionine, a metabolite produced by *Candida albicans*, activates the hypoxia-inducible factor (HIF)-2α signaling pathway in the gut by promoting the interaction between HIF-2α and HIF-1β. This activation thus increases ceramide production and accelerates atherosclerosis progression [8].

## Causality between HF and Gut Microbiota

Gut dysbiosis refers to alterations of the composition and function of the gut microbiota, triggered by dietary changes, antibiotic use, and infections [1]. Studies have identified similar microbiome disruptions in HF patients, suggesting a crucial role of the gut microbiota in HF progression and pathogenesis [9].

Nagatomo and Tang proposed the “gut hypothesis of HF”, which suggests that reduced cardiac output mediates the ischemia and disruption of the intestinal mucosa. These changes increase gut permeability, promote gut dysbiosis, and facilitate bacterial translocation, collectively contributing to inflammation in HF [10]. Reduced cardiac output in HF triggers sympathetic vasoconstriction, redirecting blood flow and causing ischemia in terminal organs, including the intestines [11]. Insufficient blood perfusion in the gut exacerbates barrier dysfunction, enabling microbiota translocation and leakage of microbial metabolites. Structurally, patients with HF often exhibit thickened intestinal walls with edema, accompanied by increased collagen content in the mucosal walls as HF severity progresses [12]. This fibrosis results from increased intestinal permeability and ischemia, which trigger inflammatory responses, promote extracellular matrix accumulation, and stimulate the proliferation of intestinal smooth muscle cells. Intestinal fibrosis not only leads to stenosis and stiffness but also impairs the absorption of HF medications, thereby exacerbating HF progression [13].

## Gut-Derived Metabolites and HF

Gut microbiota can stimulate a healthy intestinal tract to release antimicrobial compounds and competitively secure nutrients and attachment sites, thereby defending against pathogen invasion and maintaining host physiological balance [14]. However, the gut microbiome can also disrupt this balance directly or indirectly by releasing bioactive metabolites. Emerging evidence highlights the critical roles of gut-derived metabolites in the pathophysiology of HF.

### Bile acids

Primary bile acids are synthesized in the liver and excreted into the intestine, where they undergo reabsorption and are transported back to the liver through enterohepatic circulation [15]. In the terminal ileum and colon, primary bile acids that do not pass through the enterohepatic circulation are converted into secondary bile acids by gut microbiota via bile acid hydrolases and 7α-dehydroxylase [15]. In patients with HF, the ratio of primary to secondary bile acids is reduced [16]. These alterations can affect the gut microbiome, potentially contributing to HF progression.

**Figure 1.**
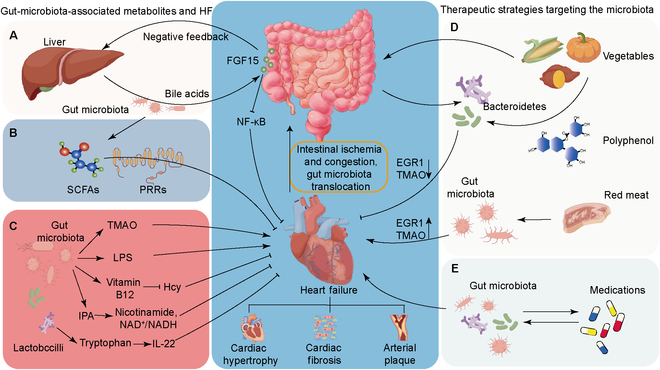
Mechanisms of gut microbiota regulation on HF. (A) Bile acids interact with FGF15 to inhibit NF-κB-mediated myocardial inflammation and fibrosis. (B) SCFAs bind to PRRs and regulate cardiac hypertrophy and fibrosis. (C) The mechanisms underlying gut microbiota-derived TMAO, LPS, vitamin B12, IPA, and IL-22 in HF. (D) A predominantly vegetarian diet reduces EGR1- and TMAO-mediated cardiac hypertrophy and fibrosis. (E) The interaction between medications and gut microbiota (details shown in Table 1). HF, heart failure; FGF15, fibroblast growth factor 15; NF-κB, nuclear factor kappa B; SCFAs, short-chain fatty acids; PRRs, pattern recognition receptors; TMAO, trimethylamine *N*-oxide; LPS, lipopolysaccharide; IPA, indole-3-propionic acid; IL-22, interleukin 22; EGR1, early growth response 1; NAD, nicotinamide adenine dinucleotide; NADH, reduced nicotinamide adenine dinucleotide.

The farnesoid X receptor (FXR) is a nuclear receptor activated by bile acids. It plays a crucial role in regulating bile acid synthesis through a negative feedback mechanism by inducing the intestinal expression of fibroblast growth factor 15 [17]. Additionally, FXR protects cardiovascular health by inhibiting nuclear factor kappa B, a key driver of inflammation and cardiac hypertrophy. Therefore, FXR-targeted therapies present a promising strategy to mitigate HF progression and improve clinical outcomes (Figure 1A).

### Short-chain fatty acids

Gut microbiota promotes fermentation processes that generate short-chain fatty acids (SCFAs) in the distal intestine, which are essential for colonic epithelial nutrition. Increased SCFA levels, particularly butyrate, have been associated with improved prognosis in individuals with HF [18]. Notably, the restoration of gut microbial composition contributed to the alleviation of inflammation and cardiac dysfunction 12 months after the initial HF diagnosis. This restoration involved an increase in beneficial bacteria and SCFAs, along with a decrease in pathogenic bacteria. In a rat model, SCFAs were shown to significantly alleviate cardiac hypertrophy and fibrosis (Figure 1B) [19].

### Trimethylamine *N*-oxide

Trimethylamine *N*-oxide (TMAO) is a metabolite produced in the liver from trimethylamine, which is generated by gut microbiota during the digestion of certain nutrients, such as choline, lecithin, and carnitine [20]. Elevated TMAO levels are associated with an increased risk of HF [21]. Fecal transplantation studies have shown that TMAO-induced proatherosclerotic phenotypes can be transferred through microbial communities (Figure 1C) [22].

### Indole-3-propionic acid

Indole-3-propionic acid (IPA) is a tryptophan-derived metabolite produced by the gut microbe *Clostridium sporogenes*. We found that IPA is reduced in heart failure with preserved ejection fraction (HFpEF) patients and a mouse model of HFpEF compared with that in controls. We further found that IPA supplementation protects against diastolic and metabolic dysfunctions in HFpEF by attenuating gut microbiota dysbiosis and enhancing cardiac nicotinamide adenine dinucleotide (NAD) salvage. IPA increases the protein levels of sirtuin 3 and decreases nicotinamide *N*-methyltransferase. These effects restore nicotinamide levels, normalize the NAD^+^/NADH ratio, and protect against diastolic dysfunction in HFpEF (Figure 1C) [23].

### Vitamin B12

Vitamin B12 is a crucial cofactor in the methionine synthase reaction, which converts homocysteine to methionine. Deficiency in vitamin B12 leads to increased homocysteine levels in tissues. High homocysteine concentrations can damage endothelial structures and impair autophagy signaling in foam cells, thereby contributing to insulin resistance, atherosclerosis, and cardiac hypertrophy, even in the absence of hypercholesterolemia (Figure 1C) [24,25].

### Vitamin A

Vitamin A (VA) plays a critical role in the development of intestinal immunity, with gut microbiota converting dietary VA into retinol [26]. Retinol-binding protein 4 (RBP4) is the primary retinol-binding protein in peripheral blood and is essential for retinol metabolism and nutrient transport. Additionally, RBP4 regulates the phenotypic transformation of vascular smooth muscle cells via the Ras homolog family member A/Rho-associated coiled-coil containing protein kinase 1 (RhoA/ROCK1) signaling pathway in atherosclerosis [27,28]. VA deficiency increases the risk of cardiovascular diseases, including arteriosclerosis, hypertension, and HF.

### Vitamin K

Menaquinones are the primary forms of vitamin K (VK), synthesized by intestinal bacteria or obtained through dietary intake [29]. VK plays a crucial role in regulating oxidative stress and inflammatory responses by participating in the γ-carboxylation of VK-dependent proteins. Additionally, VK exerts anti-inflammatory effects by inhibiting nuclear factor-kappa B signaling and enhancing growth arrest-specific 6/AXL receptor tyrosine kinase (Gas6/Axl) signaling, helping to prevent vascular calcification [30]. However, gut microbiota dysbiosis can impair VK synthesis, promoting vascular smooth muscle cell calcification, accelerating atherosclerosis progression, and increasing the risk of hypertension and cardiovascular diseases.

### Inflammation-related metabolites

The gut microbiota can maintain the balance of the host immune system through the production of inflammation-related metabolites. For instance, lipopolysaccharide induces localized intestinal inflammation and metabolic endotoxemia, producing pro-inflammatory mediators and activating inflammatory cascades. These processes contribute to the development and rupture of atherosclerotic plaques [31]. Conversely, lactobacilli can metabolize dietary tryptophan to stimulate interleukin 22 (IL-22) production. IL-22 has been shown to preserve mitochondrial membrane potential and alleviate cardiomyocyte apoptosis caused by oxidative stress [32]. Notably, IL-22 supplementation has been demonstrated to effectively prevent ventricular dysfunction and HF after myocardial infarction (Figure 1C) [33].

## Modulating the Gut Microbiota to Improve HF

Aside from host genetics, environmental factors—such as diet, medications, and medical treatments—make essential sense in modulating microorganisms. These influences contribute to individual variability in disease susceptibility and treatment responses.

### Diet

Fiber and acetate have shown protective effects on gut health by mitigating dysbiosis (lowering the Firmicutes-to-Bacteroidetes ratio) and increasing acidogenic Bacteroidetes. Acetate reduces renal fibrosis by down-regulating early growth response 1, a key regulator of cardiovascular fibrosis and inflammation in the heart and kidneys (Figure 1D) [34].

Maternal fiber intake significantly influences offspring gut microbial gene expression into adulthood, thereby reducing cardiovascular disease risk. Polyphenols, antioxidant compounds found in fruits, vegetables, and grains, enhance gut microbiota diversity and stimulate SCFA production. Diets rich in polyphenols help regulate cardiovascular factors, including blood pressure, endothelial function, and lipid levels [35]. Conversely, a red-meat-heavy diet elevates plasma TMAO levels, while a vegan diet effectively reduces them [36]. Western diets high in saturated fats and sucrose disrupt intestinal barrier function, leading to elevated TMAO production, endotoxemia, inflammation, and gut barrier dysfunction [37].

### Environmental factors

Gut microbiota is highly susceptible to environmental influences, with both extrinsic and intrinsic factors potentially impairing their composition and function [38]. Environmental factors such as heavy metals, pesticides, and artificial sweeteners can disrupt the gut microbiome. For instance, exposure to these toxic chemicals alters microbial composition and metabolite production in mice [39,40]. Heavy metals and pesticides impair gut microbiota by disrupting bacterial gene expression and metabolite secretion involved in neurotransmitter synthesis [41]. Similarly, artificial sweeteners influence bacterial metabolite pathways, leading to glucose intolerance and promoting obesity and inflammation [42].

### Exercise

Physical exercise is an effective way to maintain health and reduce the risk of cardiovascular disease [43]. Interestingly, exercise can also modulate gut microbiota composition, improving microbiota profiles and increasing the abundance of beneficial species such as *Clostridiales*, *Roseburia*, *Lachnospiraceae*, *Erysipelotrichaceae*, *Ruminococcaceae*, and *Eubacteriaceae*. These microbes produce butyrate, which helps lower hypertension by suppressing the prorenin-receptor-mediated intrarenal renin–angiotensin system [44]. Additionally, exercise increases the Bacteroidetes/Firmicutes ratio, elevating SCFA levels and reducing endotoxemia [45].

### Physiological and pathological conditions

Under physiological conditions, the intestinal flora secretes various metabolites that regulate host biological activities. However, pathological conditions, particularly those involving the digestive system or metabolism, can disrupt the composition of the gut microbiota and its metabolites, negatively impacting host health. Inflammatory bowel disease (IBD) leads to dysbiosis and reduced microbial diversity. In IBD patients, facultative anaerobes and invasive, adherent *Escherichia coli* strains proliferate rapidly, and microbiota transplantation from IBD mice induces bowel inflammation in healthy mice [46]. Similarly, type 2 diabetes and obesity alter gut microbiota composition, increase circulating inflammatory proteins, and promote chronic inflammation, thereby exacerbating cardiovascular risk [47].

### Medications

The extensive use of medications can alter the characteristics of disease-associated microorganisms, while microorganisms changes influence drug metabolism and host responses [5]. For instance, *Eggerthella lenta* can inactivate digoxin, a commonly used heart medication [48]. Here, we summarize the effects of commonly used cardiovascular medications on gut microbiota in Table 1.

**Table 1.** The impact of cardiovascular drugs on gut microbiota and their mechanisms in disease management

Cardiovascular drugs	Impact of drugs on gut microbiota	Mechanisms underlying the impact of drugs on gut microbiota
Angiotensin II receptor blockers (ARBs) [52,53]	Mitigate structural damage to both the vascular and intestinal systems	Inhibit Ang II/AT1R signaling, augmenting ACE2 activation in the Ang-(1–7)/Mas pathway
Angiotensin-converting enzyme inhibitors (ACEIs) [54]	Improve intestinal barrier function and reduce inflammatory responses	Reduce TMA entry into the circulation and increase SCFA production by improving gut microbiome health
Calcium channel blockers (CCBs) [55]	Bile acid metabolism indirectly regulates the gut microbiota	Suppress mitochondrial calcium and intestinal bile acid metabolism via the FXR/TGR5 signaling pathway
Diuretics [56]	Disrupt the balance of water, electrolytes, acid–base equilibrium, and the gut microbiota	Induce gastrointestinal congestion and intestinal wall edema, resulting in endotoxemia that triggers inflammatory responses and oxidative stress
Beta blockers [57,58]	The gut microbiota plays a crucial role in regulating the activity of extrinsic sympathetic neurons within the intestine	Compromise the intestinal barrier’s integrity by modulating the brain–gut axis
Statins [59]	Disruptions in bile acid metabolism can disturb the balance of the gut microbiota	Reduce the abundance of gut microbiota, thereby inhibiting the conversion of chenodeoxycholic acid (CDCA) to ursodeoxycholic acid (UDCA)
Antiplatelet drugs [60]	Gut microbiota indirectly influences coagulation function by regulating inflammatory responses	Regulate platelet function and reactivity through TMAO levels, thereby contributing to systemic thrombus formation
Anticoagulant drugs [61]	Regulate vitamin K metabolites	Inhibit the production of vitamin K by gut microbiota, leading to disruption in the synthesis of coagulation factors II, VII, IX, and X

Ang, angiotensin; AT1R, angiotensin II receptor type 1; ACE2, angiotensin-converting enzyme 2; TMA, trimethylamine; FXR, farnesoid X receptor; TGR5, Takeda G protein-coupled receptor 5

The gut microbiome also influences liver enzymes involved in drug metabolism, affecting circulating drug levels and therapeutic outcomes. A recent study on nearly 1,000 drugs found that 24% inhibited at least one human gut bacterium in culture, highlighting the potential for drug interactions mediated by the gut microbiome (Figure 1E) [49].

## Techniques in Gut Microbiome Research

In gut microbiome and cardiovascular research, 16S ribosomal RNA amplicon sequencing has been instrumental in tracking microbial shifts associated with disease [50]. Mass spectrometry has advanced the field by facilitating the measurement of metabolites such as TMAO, carnitine, symmetric dimethylarginine, and amino acids. Techniques like immunohistochemistry, in vitro impedance measurements, and enzyme-linked immunosorbent assays have enhanced our understanding of the gut barrier’s role in HF. RNA sequencing has provided valuable insights into TMAO’s contribution to abdominal aortic aneurysm formation [2,51]. Moreover, fecal microbiota transplantation (FMT) studies have shown improvements in cardiac function and reduced intestinal damage, indicating the strong connection between gut health and heart function. The current techniques used in gut microbiome research are outlined in Table 2.

**Table 2.** Techniques in gut microbiome research

Techniques	Advantages	Limitations
16S rRNA amplicon sequence [62,63]	High-throughput, species-level analysis of microbial communities	Limited resolution in distinguishing closely related species, potential PCR biases, sequencing errors, and contamination from host DNA
Mass spectrometry [63]	High sensitivity and specificity in metabolite detection, comprehensive compound analysis, and rapid, automated high-throughput capabilities	Large biomolecules, without tandem techniques, pose challenges in data interpretation due to the complex chemical diversity of the gut microbiome
Immunohistochemistry	Identifies bacterial species in complex gut environments and visualizes microbial spatial distribution and host interactions	Narrow detection range, risk of false positives, complex sample preparation, and subjective interpretation
ELISA	Quantitative analysis for measuring specific proteins and metabolites, offering versatility in detecting a wide range of targets	Cross-reactivity causing false positives and a limited detection range for comprehensive microbial analysis
RNA sequencing	Provides insights into active gene expression and dynamic changes within the gut microbiota	rRNA contamination; detects only actively transcribed genes and provides limited information on nonexpressed genes
Fecal microbiota transplantation (FMT) [64,65]	FMT introduces diverse microbes and provides a nonpharmacological alternative to drug therapies	Varied FMT efficacy, challenging donor selection, invasive methods, and uncertain long-term safety and risks

rRNA, ribosomal RNA; PCR, polymerase chain reaction; ELISA, enzyme-linked immunosorbent assay

## Modulating the Gut Microbiome for HF Improvement: Challenges and Prospects

Current evidence highlights the gut microbiome’s significant role in modulating HF progression, although its full therapeutic potential has yet to be realized. This uncertainty primarily due to the variability in microbial populations and individual host differences, leading to ambiguous standards for the personalized modulation of the gut microbiome. This emphasizes the necessity of individualized and targeted approaches in microbiome adjustment. Future studies should therefore focus on assessing how HF medications affect gut microbial populations and metabolites, as well as identifying targeted microbial interventions to enhance HF outcomes. This approach could lay a strong foundation for integrating microbiome modulation with existing HF treatments, optimizing therapeutic efficacy.

Most research to date has examined correlations between probiotic intake and HF phenotypes, but relatively few studies have explored how prebiotic or probiotic interventions specifically alter the microorganism flora and function. This gap suggests that microbiomes may influence HF treatment outcomes, warranting further investigation into the underlying mechanisms.

Additionally, factors such as HF classification, disease stage, comorbid conditions, dietary habits, drug interactions, physical health, and psychological status should be considered when modulating the gut microbiome for HF patients. These considerations could help develop more individualized treatment strategies. Given that current research primarily involves animal models, which may not capture the complexity and heterogeneity of human HF—including its comorbidities, diverse etiologies, and lifestyle variations—there is an urgent need for well-designed, large-scale, prospective cohort studies to provide strong, evidence-based medical insights.

## Concluding Remarks

Integrating traditional HF therapies with innovative gut microbiome modulation offers a promising therapeutic direction. However, applying these strategies in clinical practice remains challenging due to individual patient differences and varying HF pathophysiology. Continued research into the biological mechanisms connecting gut microbiota with HF therapies is essential, as it may yield new biomarkers and more effective treatment protocols that enhance patient quality of life and clinical outcomes. As evidence grows, this integrative approach has the potential to transform HF management, providing more personalized and effective interventions tailored to patient-specific needs.
